# Connectivity from the ventral anterior cingulate to the amygdala is modulated by appetitive motivation in response to facial signals of aggression

**DOI:** 10.1016/j.neuroimage.2008.07.045

**Published:** 2008-11-15

**Authors:** Luca Passamonti, James B. Rowe, Michael Ewbank, Adam Hampshire, Jill Keane, Andrew J. Calder

**Affiliations:** a Medical Research Council, Cognition and Brain Sciences Unit, 15 Chaucer Road, Cambridge, CB2 7EF, UK; b National Research Council, Institute of Neurological Sciences, Piano Lago di Mangone (CS), 87050, Italy; c Department of Clinical Neurosciences, University of Cambridge, Cambridge, CB2 2QQ, UK; d Medical Research Council Behavioural and Clinical Neurosciences Institute, Downing Site, Cambridge, CB2 3E, UK

**Keywords:** Aggression, Behavioural approach/behavioural inhibition system, Individual differences, Effective connectivity, fMRI, Emotion regulation

## Abstract

For some people facial expressions of aggression are intimidating, for others they are perceived as provocative, evoking an aggressive response. Identifying the key neurobiological factors that underlie this variation is fundamental to our understanding of aggressive behaviour. The amygdala and the ventral anterior cingulate cortex (ACC) have been implicated in aggression. Using functional magnetic resonance imaging (fMRI), we studied how the interaction between these regions is influenced by the drive to obtain reward (reward–drive or appetitive motivation), a personality trait consistently associated with aggression. Two distinct techniques showed that the connectivity between the ventral ACC and the amygdala was strongly correlated with personality, with high reward–drive participants displaying *reduced negative* connectivity. Furthermore, the direction of this effect was restricted from *ventral ACC to the amygdala* but not *vice versa*. The personality-mediated variation in the pathway from the ventral anterior cingulate cortex to the amygdala provides an account of why signals of aggression are interpreted as provocative by some individuals more than others.

## Introduction

For the majority of individuals, facial signals of aggression are threatening, inducing anxiety or fear. For a smaller but significant proportion of society they are interpreted as provocative, evoking an aggressive response. Identifying the neurobiological factors that underlie this variation is one key factor in understanding of aggression. A large body of research has demonstrated that a personality trait linked to the motivation to gain reward (reward–drive or appetitive motivation) is associated with altered perception of facial signals of aggression, clinical disorders of aggression, and heightened tendency to experience and display anger but not other negative emotions, such as fear (Carver, 2004; Cole and Zahn-Waxler, 1992; Cooper et al., 2008; Diefendorff and Mehta, 2007; Gray, 1994; Harmon-Jones, 2003, 2004; Putman et al., 2004; Smits and Kuppens, 2005). High reward–drive individuals also show increased attention to facial signals of aggression (Putman et al., 2004), attributed to their increased tendency to interpret them as signals of provocation or social challenge (Beck, 1976; van Honk et al., 2001); the same effect has also been observed in high trait anger participants (van Honk et al., 2001).

In recent research we have explored the neural basis of this effect. Functional Magnetic Resonance (fMRI) data have shown that reward–drive selectively modulates the responses of the amygdala and ventral anterior cingulate (ACC) to viewing facial signals of aggression (Beaver et al., 2008). These regions have a wider role in emotion regulation, but their contribution to aggression has been highlighted by clinical and comparative research (Blair, 2003; Coccaro et al., 2007; Davidson et al., 2000; Nelson and Trainor, 2007). Experience of anger has also been associated with increased amygdala and decreased ventromedial prefrontal/ventral ACC activation (Dougherty et al., 2004). Hence, it is of interest that viewing facial signals of aggression produces an analogous pattern (relative to neutral or sad expressions) that becomes more pronounced in high reward–drive individuals (Beaver et al., 2008).

However, critical questions remained unanswered. Our previous study did not address potentially important interactions that occur during the processing of aggressive displays. The amygdala and the ventral ACC are anatomically connected (Aggleton, 1985; Ghashghaei et al., 2007). Thus, formal analyses of “effective connectivity” would allow heightened understanding of the network processing signals of aggression in humans, and the manner in which it is modulated by personality traits such as the reward–drive (appetitive motivation). We investigated these issues in a new experiment that focused on the neural correlates of viewing angry and neutral expressions.

Our study had three aims. First to determine whether viewing facial signals of aggression relative to neutral expressions is associated with a significant change in connectivity. Second, there is converging evidence demonstrating that the ventral ACC influences the amygdala more than *vice versa* (Etkin et al., 2006; Ghashghaei et al., 2007; LeDoux, 2003; Quirk et al., 2003; Rosenkranz and Grace, 2002). We therefore addressed whether connectivity effects were focused on the top-down influence of the ventral ACC on the amygdala, rather than *vice versa* or a bidirectional effect. Third, to determine whether these effects were modulated by individual variation in reward–drive.

To test these hypotheses we employed different but complementary approaches to the analysis of fMRI data from healthy human participants while they viewed angry or neutral faces. First we tested for PsychoPhysiological Interactions (PPI) using General Linear Models (GLM) (Friston et al., 1997). Then we addressed the directions of causal connections with Dynamic Causal Modeling (DCM) (Friston et al., 2003). In both cases we tested the extent to which the effects were modulated by reward–drive.

## Subjects and methods

### Participants

Twenty-one right-handed healthy volunteers participated in the study (10 females; age range = 19–39; mean age = 25.3 years; mean IQ = 120, SD = 21.6) with normal, or corrected to normal vision. The study was given a favourable opinion by the Local Research Ethics Committee for Cambridge and all participants provided written informed consent. Participants with a history of neurological or psychiatric disease or currently taking medication affecting the Central Nervous System were excluded from the study.

Participants completed the Behavioural Approach/Behavioral Inhibition Scales (BAS/BIS) before scanning (Carver, 2004). This questionnaire consists of three subscales that measure sensitivity to reward—the motivation to gain reward (BAS–reward–drive), the willingness to approach a potentially rewarding event on the spur of the moment (BAS–fun seeking behaviour), and the positive reactions to the occurrence or anticipation of reward (BAS–reward sensitivity). An additional scale measures responsiveness to punishment (BIS). We were primarily interested in the reward–drive subscale because this is more predictive of aggressive behaviour than other BAS or BIS measures (Carver, 2004; Harmon-Jones, 2003).

In light of the potential for angry faces to represent social signals of threat to anxious individuals (Mogg and Bradley, 1998), participants also completed both the Spielberger State and Trait Anxiety Inventory (STAI) (a measure of the “transitory” (state) and “relatively stable” (trait) tendency to respond with anxiety to perceived threats in the environment) (Spielberger et al., 1983) and the brief version of the Fear of Negative Evaluation scale (FNE-Brief) (a measure of “apprehension about others' evaluations” and “distress over social negative evaluations”) (Collins et al., 2005). Finally, to exclude any possible confound by concomitant depressive symptoms, participants also completed the Center for Epidemiologic Studies Depression Scale (CES-D Scale), a self-report questionnaire which has been developed as a screening measure in the general population (Sawyer-Radloff, 1977).

### fMRI task

**Fig. 1:**
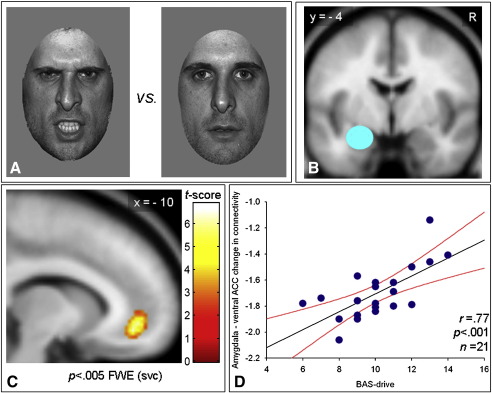
(A) Example faces during the gender discrimination task. Either an angry or neutral face was presented on each trial. (B) Source region for the PsychoPhysiological Interaction (PPI) in the General Linear Model (GLM). The left amygdala was defined as a 10 mm sphere using two different approaches (see PPI GLM Experimental Procedures for details). The slice shown is at *y* = − 4 mm in MNI space (Montreal Neurological Institute). R: right side. (C) PPI GLM Statistical Parametrical Map (SPM). This SPM {t} map for the higher order PPI demonstrates that the ventral Anterior Cingulate Cortex (ACC) is connected with the amygdala (source region) as a function of the angry context *and* of the reward–drive (appetitive motivation) personality. Color bar represents *t* statistics (see the PPI GLM Results section for details). FWE: Family Wise Error, small volume correction (svc) (see the PPI GLM Results section for details). The slice shown is at *x* = − 10 in MNI space (Montreal Neurological Institute). The whole-brain map is thresholded at *p* < .001, uncorrected. (D) Data plot for the PPI showed in the panel C. There is a highly statistically significant correlation (*r* = .77, *p* < .001) between the PPI (i.e. the amygdala-ventral ACC connectivity as function of the angry context) and the individual differences in reward–drive score with participants scoring *lower* presenting the more *negative* connectivity as opposed to individuals scoring *highe*r displaying the less *negative* values. The regression line (black) and the 95% confidence intervals (red lines) are shown. BAS–drive: Behavioral Approach System–drive subscale (reward–drive or appetitive motivation).

After pre-training, participants underwent functional MRI scanning. They performed a gender discrimination task while viewing grey-scale photographs of angry or neutral faces (Fig. 1A). These were presented by an angled mirror above the participants’ eyes, which reflected images back-projected onto a translucent screen in the bore of the magnet behind the participant's head. The facial expression stimuli were selected from the NimStim Face Stimulus Set (www.macbrain.org) and Karolinksa directed emotional faces (KDEF) on the basis of independent emotional ratings. There were 30 different identities (half female) for each expression. Stimuli were presented in alternating 21-s epochs each containing six stimuli from the one category (angry or neutral) intermixed with six null events. Eighteen epochs of each category were presented.

Each face trial comprised a 1000 ms presentation of a face followed by a low contrast central cross (750 ms). The participants categorised the gender of each face within the 1750 ms trial period. Null events constituted a 1750 ms presentation of the same low contrast central cross. The gender and identity of the faces were fully randomized. The stimuli during each epoch were also pseudo-randomized with respect to trial type (face or null), such that no more than three consecutive trials were of the same format (face or null). Pseudorandomization enhanced design efficiency while preserving the unpredictability of stimulus onsets in naïve participants. Reaction times and accuracy were recorded. The total experiment duration was 12 min and 36 s.

### Image acquisition and preprocessing

MRI scanning was performed on a 3-Tesla Trio Tim Magnetic Resonance Imaging scanner (Siemens, Germany) with a head coil gradient set at the Medical Research Council Cognition and Brain Sciences Unit. Whole-brain data were acquired with echo-planar T2⁎-weighted imaging (EPI), sensitive to BOLD signal contrast (46 coronal slices, 3 mm-thickness; TR = 2800 ms; TE = 30 ms; flip angle = 78°; FOV 192 mm; voxel size: 3 × 3 × 3 mm). The first 3 volumes were discarded to allow for equilibration effects. T1 weighted structural images were acquired at a resolution of 1 × 1 × 1 mm.

Data were analyzed using SPM5 software (www.fil.ion.ucl.ac.uk/spm/). The EPI images were sinc interpolated in time to correct for slice time differences and realigned to the first scan by rigid body transformations to correct for head movements. The mean EPI was computed for each participant and inspected to ensure that none showed excessive signal dropout in medial temporal and ventromedial/ventral anterior cingulate cortices. EPI and structural images were coregistered and normalized to the T1 standard template in MNI space (Montreal Neurological Institute (MNI) – International Consortium for Brain Mapping) using linear and non-linear transformations, and smoothed with a Gaussian kernel of full-width-half-maximum (FWHM) 8-mm.

### Analysis of regional effects

Our aim was to assess connectivity between the amygdala and other potential brain ‘target’ regions during the processing of angry faces and to study any possible higher order interaction with the reward–drive (appetitive motivation) personality trait. Therefore, we first sought to obtain an amygdala reference region to use as the ‘source’ region for psychophysiological interactions (PPI) (see PPI GLM methods for details). To this end, a random effects model was implemented using a two stage process, of within (first level) and between (second level) participants modelling in turn. This random-effects analysis assessed effects on the basis of inter-participant variance and thus allowed inferences about the population that the participants were drawn from. For each participant we used a General Linear Model (GLM) to assess regionally specific effects of task parameters on BOLD indices of activation. The model included three experimental factors (angry faces, neutral faces, and central cross) and effects of no interest (realignment parameters) to account for motion-related variance. Low-frequency signal drift was removed using a high-pass filter (cutoff 128 s) and an autoregressive model (AR1) was applied to adjust for autocorrelations.

The individual first level images were generated using the contrast of angry *vs.* central cross, rather than angry *vs.* neutral contrast. This comparison increased the power to functionally detect the amygdala given that neutral faces have also been shown to activate the amygdala (Fitzgerald et al., 2006; Wright and Liu, 2006). However, the angry *vs.* central cross comparison was only used to functionally define the amygdala coordinates as the source region for PPI. We always employed the angry *vs.* neutral faces comparison in the connectivity analyses in accordance with the main hypotheses of the study (see specific PPI GLM and DCM methods section).

### Effective connectivity analyses

#### Psycho-Physiological Interaction in a General Linear Model (PPI GLM)

The physiological connectivity between two regions may vary with the psychological context (Friston et al., 1997). In our study, the connectivity arising from face presentation is modulated by the context of angry *vs.* neutral faces. This constitutes a psychophysiological interaction (PPI) (Friston et al., 1997). We sought to identify ‘target’ regions which had such differential connectivity with the source region (amygdala) according to the context (anger *vs.* neutral). This was achieved using a moderator variable, derived from the product of source activation and context. In this way, regions are identified not because of correlation with the amygdala activation or the presence/absence of angry faces, but because of the interaction between these two variables.

The amygdala response to anger *vs*. central cross was restricted to the left hemisphere (see Supplementary Table 1). Hence, for the subsequent functional connectivity analyses, only the left amygdala was considered as the source region (Fig. 1B). For each participant, we computed the angry *vs*. neutral faces contrast to determine the local maximum that was the nearest voxel to the activation peak in the left amygdala defined by the whole group cluster (Supplementary Table 1). Next, a 10-mm sphere was constructed around this participant-specific local maximum and the time-series for each participant computed using the first eigenvariate from all voxels’ time series. Using this approach the centre of the amygdala ROI for each participant was the most significant voxel, meaning that the centre of the amygdala ROI was slightly different across participants. So in a separate analysis we also employed a standardised 10-mm sphere across all participants (centre for all participants: *x* − 24, *y* − 4, *z* − 12 which was the maximal voxel for the anger *vs.* central cross contrast, see Supplementary Table 1). Again, the time-series for each participant was computed by using the first eigenvariate from all voxels’ time series in this common left amygdala ROI. Regardless of the approach used to extract the time-series in the source region we obtained highly consistent results (see PPI GLM results section).

The BOLD time series for each participant was deconvolved to estimate a ‘neuronal time series’ for this region (Gitelman et al., 2003). The psycho-physiological interaction term (PPI regressor) was calculated as the element-by-element product of the left amygdala neuronal time series and a vector coding for the main effect of task (1 for angry faces, − 1 for neutral faces, and 0 for null events). This product was re-convolved by the canonical hemodynamic response function (hrf). The model also included the main effects of task convolved by the hrf, and the movement regressors as effects of no interest. Participant specific PPI models were run, and contrast images generated for positive and negative PPIs. The identified regions have greater or lesser connectivity with the source region according the context of angry *vs.* neutral face presentation.

The 21 contrast images were entered into second level GLM analyses for contrasts of interest, and SPM-maps generated using Gaussian Random Field theory to make statistical inferences (Friston et al., 1995). To test regions with changes in connectivity with the source region following angry *vs*. neutral faces context in the whole sample (regardless of any personality dimension) we used a one sample *t* test. To identify regions for which the changes in connectivity with the source region (following angry *vs.* neutral faces context) were correlated with the individual variation in reward–drive score, we employed a regression model within SPM. Using distinct regression models, we also explored any correlations with the fun seeking and reward responsiveness subscales, although these dimensions have been associated with measures of aggression to a lesser extent (Carver, 2004; Putman et al., 2004). Moreover, as the neural response to angry faces has also been found to be influenced by anxiety and depression (Ewbank et al., submitted; Leyman et al., 2007; Phan et al., 2006), we investigated any potential effect of the participants' STAI (state and trait anxiety) scores, FNE (fear negative evaluation) scores and CES-D (depression) scores on the connectivity between the amygdala and other potential brain ‘target’ regions (regression models).

Two approaches to statistically threshold maps were applied. First, for small volume corrections (svc) within *a priori* regions of interest (ROI), the threshold was set at *p* < .05 Family Wise Error (Worsley et al., 1996). For the effect of reward–drive, we defined a 15-mm sphere in the ventral ACC ROI using as center the local maxima derived from our previous study (*x* − 15, *y* 36, *z* − 12) (Beaver et al., 2008). For the effect of anxiety the ROIs comprised the dorsal ACC (MNI local maxima: *x* − 2, *y* 12, *z* 40) and the ventrolateral prefrontal cortex (VLPFC) (MNI local maxima: *x* − 36, *y* 16, *z* − 6), defined from previous work (Bishop et al., 2004; Bishop et al., 2007). Second, to explore other possible regions which were not predicted, a threshold of *p* < .001, uncorrected was used.

#### Dynamic Causal Modelling (DCM)

To understand further the effective connectivity between the amygdala and the ventral ACC (the two regions showing a higher order interaction with the reward–drive personality, see the PPI GLM results section) we used dynamic causal modelling (DCM) (Friston et al., 2003). DCM enables an alternative method of analysis of psychophysiological interactions within a hypothesis driven anatomical model. More specifically, the DCM explains regional effects in terms of changing patterns of connectivity amongst regions according to experimentally induced contextual modulation of connection strengths. The principal advantage of DCM over the GLM implementation of PPI analysis is the ability to make inferences about the directionality of causal connections.

The DCM anatomical model was built from specific hypotheses about the amygdala and the ventral ACC as key neural structures for the processing and recognition of emotional faces (Adolphs et al., 1999; Broks et al., 1998; Calder et al., 2001; Hornak et al., 2003). A 10 mm sphere ROI was created in the left amygdala by using the local maximum for each participant that was the nearest voxel to the activation peak in the left amygdala defined by the whole group cluster (Supplementary Table 1). For the ventral ACC, a 15 mm sphere ROI was defined using the local maxima for each subject that was the nearest voxel to the activation peak identified by the PPI analysis (see Results section, Analysis of effective connectivity 1: PPIs in the General Linear Model).

**Fig. 2:**
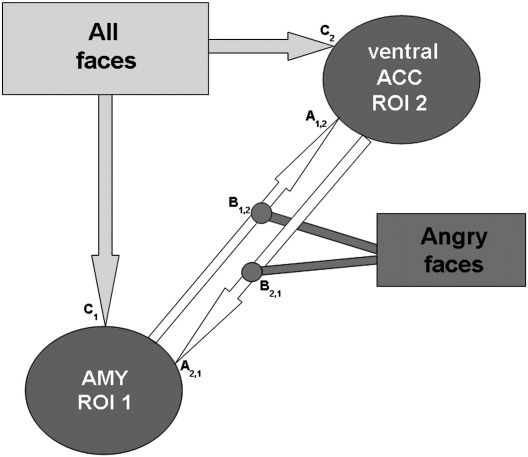
Dynamic Causal Modeling (DCM) neural network. The amygdala (AMY) and the ventral anterior cingulate cortex (ACC) are the regions of interest (ROIs). The faces (regardless of the emotion) were entered as driving input (DCM matrix C values) directly in both the ROIs according with electrophysiological data. The intrinsic connectivity between ROIs (DCM matrix A values) was modelled as bidirectional (see DCM methods section for further details). ‘Angry faces’ was used as bilinear modulator (DCM matrix B values) of connectivity in both pathways (from ventral ACC to AMY and from AMY to ventral ACC).

The intrinsic connections (connectivity regardless the main effect of the task, DCM Bilinear matrix A value; see Fig. 2) were modelled as bidirectional in accord with anatomical evidence showing that the ventral ACC projects to the amygdala and *vice versa* (Aggleton et al., 1980; Amaral and Price, 1984; Ghashghaei et al., 2007; McDonald and Mascagni, 1996).

The modulation by the emotion of faces was included as a bilinear effect expressing the contextual moderator (i.e. anger versus neutral context; DCM Bilinear matrix B value; see Fig. 2). A significant effect of the bilinear variable on connectivity indicates a PPI (Friston et al., 2003).

**Supplementary Figure 1:**
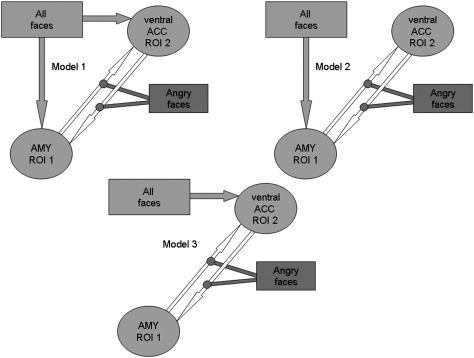
Different DCM models including the ventral Anterior Cingulate Cortex (ACC) and the Amygdala (AMY) as region of interest (ROI). The three models differ each other with respect to where the driving inputs (all faces, regardless of emotional expressions) were injected: both in the ventral ACC and in the AMY (model 1); only in the AMY (model 2) and only in the ventral ACC (model 3). DCM: Dynamic Causal Modelling.

We first tested three different DCM models in which the driving inputs (i.e. presentation of faces regardless of the emotional expression) were ‘injected’ into different parts of the network (Supplementary Fig. 1). This ‘injection’ determines the origin of perturbation of the network, from which other points in the network will be activated according to the pattern of connectivity. For the first “parallel” model (Fig. 2), face driving inputs (i.e., all faces, regardless of emotional expressions) were ‘injected’ into both amygdala and the ventral ACC. This was considered the more neurobiologically plausible model according to electrophysiological literature in animals (Leonard et al., 1985; Rolls, 2007) and humans (Eimer and Holmes, 2007; Eimer et al., 2003; Kawasaki et al., 2001; Oya et al., 2002) suggesting that the amygdala and ACC respond very quickly and within approximately the same time-scale window (∼ 110–220 ms) to faces. The two last “serial” models differ from the first one with respect to where the driving inputs were injected: only in the amygdala (model 2) or only in the ventral ACC (model 3). When comparing all models using Bayesian model selection implemented within SPM5 software we assumed that all of them were equally likely *a priori* (Penny et al., 2004). We used the selection procedure which estimates the probability of each model given the data using Akaike's information criterion (AIC) and Bayesian's information criterion (BIC) approximations to each model's log-evidence or marginal likelihood (Penny et al., 2004).

**Supplementary Figure 2:**
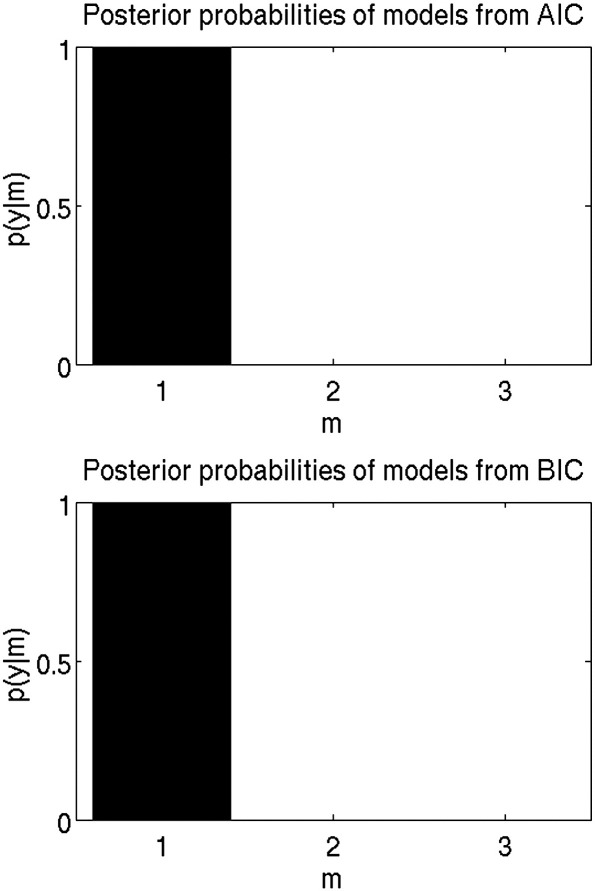
Comparison between the three DCM models showed in the Supplementary Fig. 1. We compared the three models using Bayesian model selection. The selection procedure estimates the probability of each model given the data using Akaike's information criterion (AIC) and Bayesian's information criterion (BIC) approximations to each model's log-evidence or marginal likelihood. We found very strong evidence, determined separately using AIC and BIC, in favour of the model 1; m (1, 2, 3): model 1, 2 and 3 according to the Supplementary Fig. 1.

For every participant we found very strong evidence (see DCM results and Supplementary Fig. 2), in favour of the “parallel” model 1. Therefore using this model we further analyzed the impact of the driving input (all faces) (DCM matrix C value: effect of faces regardless of the expression) on both ROI activities, the intrinsic connectivity between the ROIs (DCM matrix A value: connectivity regardless the main effect of the task), and the modulatory effect by emotion (DCM matrix B value: angry *vs.* neutral context) on specific bidirectional intrinsic connections (from amygdala to the ventral ACC and from the ventral ACC to amygdala) in each participant at a fixed-effects level.

One sample *t* tests were performed on the A-, B- and C-DCM matrix values to enable inference about the whole group (irrespective of any personality scores). Finally, individual B-, and C-DCM matrix values were entered into simple regression models with reward–drive, STAI (state or trait) anxiety, FNE (fear negative evaluation), and CES-D (depression) scores as main regressors in order to identify any specific modulation by the reward-drive personality, anxiety or depression.

## Results

### Participants

The scores on the BAS/BIS subscales, on the Spielberger State and Trait anxiety (STAI), on the Fear of Negative Evaluation scale (FNE-Brief), and on the Center for Epidemiologic Studies Depression Scale (CES-D) were as follows: BAS reward-drive, range 6 to14 (0.5–80 percentile of the normal population), mean = 10.09, SD = 2.02; BAS-reward responsiveness, range 12 to 20 (0.4–87 percentile), mean = 16.66, SD = 1.85; BAS-fun seeking, range 7 to15 (0.8–87 percentile), mean = 11.66, SD = 2.29; BIS, range 17 to 28 (14–98 percentile), mean = 21.71, SD = 3.54; State anxiety, range 20 to 43 (1.7–94 percentile), mean = 30.71, SD = 6.76; Trait anxiety, range 21 to 52 (2.5–99 percentile), mean = 36.52, SD = 8.39; FNE range 0 to 42 (0–94 percentile), mean = 20.45, SD = 12.73; CES-D, range 1 to 24 (17–96 percentile), mean = 11, SD = 6.20.

We found a borderline negative correlation between the reward–drive and BIS measures (*r* = − .40, *p* = .06). Thus, to exclude any contribution from BIS scores, they were included as a covariate of no interest in the general linear model (GLM).

### fMRI behavioural

Across the whole group, reaction times (RT) during the gender decision task were longer for angry (mean RT = 717 ms, SD = 88) than for neutral face trials (mean RT = 696 ms; SD = 74; *t* (20) = 2.46, *p* < .02). The difference in RT between angry and neutral face trials correlated negatively with reward–drive scores (*r* = − .51, *p* < .02), reflecting faster gender categorization of angry faces with increasing reward–drive. This accords with previous research showing increased attention to angry faces in high reward–drive individuals (Putman et al., 2004). Again, to exclude any confounding effect of the response time, the differences in RT between angry and neutral faces trials were factored out in the PPI GLM model.

There were no correlations between differences in RT and other BAS/BIS subscales, measures of anxiety, or depression scores (*r*s < .23, *p*s > .15). In addition, task accuracy was consistently high across participants (mean accuracy = 94.5%, SD = 2.39) with no statistically significant correlations with any of the BAS/BIS subscales, measures of anxiety, or depression scores (*r*s < .12, *p*s > .48).

### Analysis of effective connectivity 1: PPIs in the General Linear Model

**Supplementary Figure 3:**
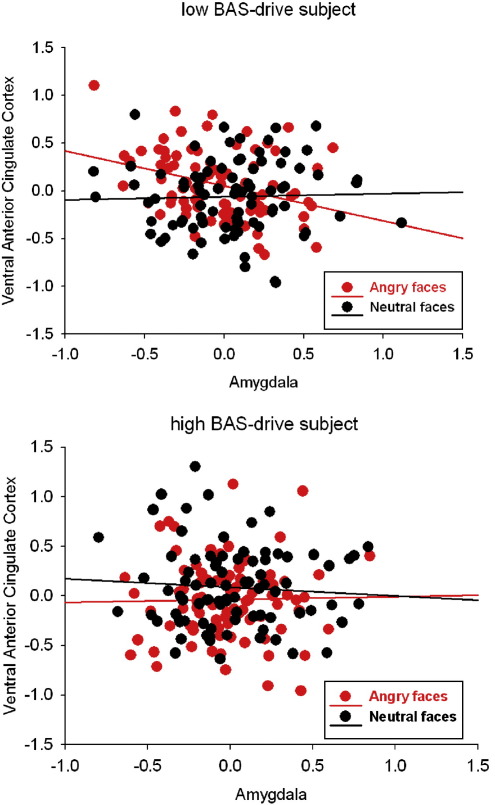
Data plot from a low BAS–drive (upper panel) and a high BAS–drive subject (lower panel). In the low BAS–drive subject, there is minimal correlation between the amygdala and ventral ACC for neutral faces, but a negative correlation for angry faces. In the high BAS–drive subject, this negative correlation for angry faces is no longer seen. For illustrative purposes, we have categorised data points to one or other condition, using a 6 s shift in the transitions between conditions to reflect the delay to peak BOLD response (after Stephan et al., Science 2003). The formal modelling of the psychophysiological interaction in general linear models incorporates the full temporal profile of the evoked hemodynamic response function (accounting for the temporal delay). BAS–drive: Behavioural Approach System–drive subscale for reward–drive or appetitive motivation.

The PPI GLM showed a borderline negative connectivity between the left amygdala (source region) and the left ventral ACC across all participants (regardless any personality dimension) that did not meet the a priori threshold for significance (*x* − 8, *y* 44, *z* 4; *t* = 2.89; *p* < .005, uncorrected) (one sample *t* test). However, our hypothesis was that the magnitude of this effect might reflect systematic individual differences in reward–drive personality, representing a higher-order PPI. As predicted, the statistical parametrical map (SPM) of this higher-order PPI again identified the left ventral ACC and was highly significant (*x* − 10, *y* 42, *z* − 10; *t* = 6.03; *p* < .005, Family Wise Error (FWE), small volume corrected (svc); Fig. 1 C). Moreover, it is striking that the left ventral ACC was one of only three regions that showed connectivity with the amygdala as a function of the anger context *and* the reward–drive personality (*x* − 10, *y* 42, *z* − 10; *t* = 6.03; *p* < .001, uncorrected). The others regions were the dorsolateral prefrontal cortex (*x* 26, *y* 34, *z* 34; *t* = 4.10; *p* < .001, uncorrected) that has been also implicated in aggression although to a lesser extent (Davidson et al., 2000), and the parietal cortex (*x* 12, *y* − 52, *z* 74; *t* = 4.68; *p* < .001, uncorrected). Anger-related changes in the connectivity between the amygdala and the ventral ACC were highly correlated with the reward–drive scores (*r* = .77, *p* < .001), ranging from *more negative* connectivity with *lower* reward–drive scores to *less negative* connectivity for *higher* reward–drive scores (Fig. 1 D, see also Supplementary Fig. 3).

In the previous analysis, the time series for the source region was extracted from a participant-specific local maximum in the left amygdala ROI, consistent with previous studies (Stephan et al., 2003). However, it is of note that we also obtained consistent results using a different approach in which the time series for each participant was extracted using the same center of the 10 mm sphere ROI for all participants (see PPI-GLM methods for details). Again, the left ventral ACC showed connectivity with the amygdala as a function of both the anger context *and* the reward–drive personality (*x* − 12, *y* 40, *z* − 10; *t* = 4.80; *p* < .02, FWE svc).

In both PPI analyses we did not find any higher-order PPI between the amygdala–ventral ACC connectivity and other BAS/BIS measures (reward responsiveness, fun seeking or BIS) (no suprathreshold voxels even reducing the threshold at *p* < .005, uncorrected). We also tested for brain ‘target’ regions showing changes in connectivity with the amygdala as a function of the trait or state anxiety and we identified different areas, including the dorsal ACC (*x* − 10, *y* 24, *z* 42; *t* = 4.27; *p* < .05, FWE, svc) and the ventrolateral prefrontal cortex (VLPFC) (*x* − 32, *y* 24, *z* − 12; *t* = 4.11; *p* < .05, FWE, svc), which have been previously associated with individual differences in anxiety (Bishop et al., 2004; Bishop et al., 2007). The anger-related changes in the connectivity between the amygdala and the dorsal ACC and the VLPFC were positively correlated with state anxiety (*r* = .69, *p* < .001 for the dorsal ACC; *r* = .65, *p* < .001 for the VLPFC), ranging from *more negative* connectivity with *lower* anxiety scores to *less negative* connectivity for *higher* anxiety scores. No significant higher-order PPI was found with the FNE (fear negative evaluation) scale (no suprathreshold voxels even reducing the threshold at *p* < .005, uncorrected). However, we identified the left ventral putamen as a region coupled with the left amygdala as a function of the CES-D (depression) score (*x* − 12, *y* 8, *z* − 10; *t* = 5.21; *p* < .0001, uncorrected; *r* = .63, *p* < .005).

In summary, the anger-related modulation of connectivity between the amygdala and the ventral ACC was strongly correlated with individual differences in reward–drive but not with other emotional dimensions (fun-seeking, reward-responsiveness, BIS, state and trait anxiety, FNE, and depression). Moreover, by covarying out any contribution by RT's and BIS scores, we have demonstrated that these variables do not account for the statistically significant effect of the reward–drive personality on amygdala-ventral ACC PPI.

### Analysis of effective connectivity 2: Dynamic causal modeling

**Fig. 3:**
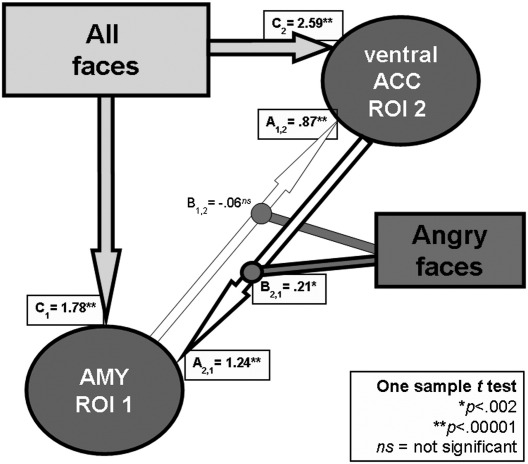
Summary of the DCM results from the whole group. The picture shows the DCM matrix mean values (**A, B, and C**) obtained from 21 participants (one sample *t* test). Faces *per se* (DCM matrix **C** values—regardless of the emotional expression) have a very strong impact on the activity of both the amygdala (AMY, ROI number 1) and the ventral anterior cingulate cortex (ACC, ROI number 2). The intrinsic connectivity (DCM matrix **A** values—regardless of the emotional expression) was highly significant in both directions. In contrast, the main effect of the emotional expression (DCM Bilinear matrix **B**—angry *vs.* neutral context) selectively enhanced the connectivity from ventral ACC to AMY (**B**_**2,1**_) but not from AMY to ventral ACC (**B**_**1,2**_). DCM: Dynamic Causal Modeling. ROI: region of interest.

Using the Bayesian model selection procedure (Penny et al., 2004), we found strong evidence that the parallel model 1 was associated with the highest probability to explain the data (see Supplementary Fig. 2). Therefore we used model 1 to test for modulatory influence of anger (*vs.* neutral) expression on each specific pathway (from ventral ACC to amygdala and from amygdala to ventral ACC). As predicted, face presentation (regardless of the emotional expression: DCM matrix C) had a very strong influence on the neural activity of both amygdala and ventral ACC ROIs in all participants (*p*s < .00001) (Fig. 3). Similarly, the intrinsic connectivity (DCM matrix A) between the ROIs in the whole sample was highly significant in both directions (from the ventral ACC to amygdala and *vice versa*) (*p*s < .00001) (Fig. 3).

The main effect of the emotional expression (angry *vs.* neutral context: DCM Bilinear matrix B) enhanced the connectivity from ventral ACC to amygdala (*p* < .002) but not *vice versa* (*p* > .2) across individuals (one sample *t* test, irrespective of the reward–drive personality) (Fig. 3). The direct comparison between the DCM Bilinear matrix B's (B values from ventral ACC to amygdala *vs.* B values from amygdala to ventral ACC) confirmed that the anger-related changes in connectivity between the ventral ACC and amygdala were not symmetrical (paired *t* test, *t*(20) = 2.54, *p* < .02).

**Fig. 4:**
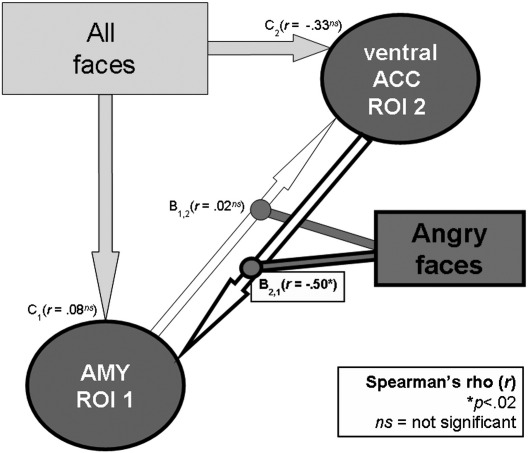
Summary of the correlations between the DCM matrix mean values (B, C) and the individual BAS–drive scores (reward–drive or appetitive motivation) (Spearman's *r*). There is no significant relationship between reward–drive scores and individual values of the effect of faces *per se* on both the amygdala and the ventral anterior cingulate cortex (DCM matrix **C** values — regardless of the emotional expression). In contrast, there is a statistically significant higher order negative correlation between the reward–drive score and the subject-specific effect of emotion (DCM Bilinear matrix **B** — angry *vs.* neutral context) on connectivity in the pathway from the ventral Anterior Cingulate Cortex (ACC) to amygdala (**B**_**2,1**_) but again (as in the one sample *t* test, see Fig. 3) not from AMY to ventral ACC (**B**_**1,2**_). DCM: Dynamic Causal Modeling. ROI: region of interest.

Of particular interest, we found a significant higher-order interaction between the reward–drive scores and the subject-specific effect of emotion on connectivity (DCM Bilinear matrix B: angry versus neutral context). There was a negative correlation between the reward–drive and the DCM bilinear moderator term (DCM Bilinear matrix B) for the connectivity in the specific pathway from the ventral ACC to the amygdala (*r* = − .50, *p* < .02) (Fig. 4). The reverse connection (from the amygdala to the ventral ACC) did not correlate with reward–drive (*r* = .02, *p* > .90). Furthermore, there was no significant influence of reward–drive on the driving inputs (presentation of faces regardless of the emotional expression: DCM Bilinear matrix C) to the amygdala or the ventral ACC (Fig. 4) and we found no correlations between STAI (state or trait anxiety), FNE (fear negative evaluation) or the CES-D (depression) scores and the B- or C-DCM Bilinear matrix values (*r*s < .21, *p*s > .36).

The specificity of reward–drive effect in the pathway from the ventral ACC to the amygdala was confirmed by comparing the *r* value derived from the correlation with reward–drive with the *r* values derived from the correlations with other personality dimensions (*t*s > 2.7, *p*s < .05, Hottelling's t tests). This means that the anger-related effective connectivity from the ventral ACC to amygdala is the only critical pathway influenced by the reward–drive personality but not by other emotional dimensions, such anxiety or depression. Group statistics for DCM results are summarized in Figs. 3 and 4 (one sample *t* tests and correlation with reward–drive scores).

## Discussion

We have shown that individual differences in reward–drive (appetitive motivation) strongly modulate the neural connectivity between the ventral ACC and amygdala while viewing facial signals of aggression. This was addressed using two complementary methods—Psychophysiological Interactions (PPI) in a general linear model (GLM) and Dynamic Causal Modelling (DCM). PPI analyses have the advantage of being anatomically unconstrained, providing an objective, data-driven approach. The PPI analysis in the whole sample (regardless of individual differences in reward–drive) identified the ventral ACC as showing a weak change in connectivity with the amygdala according to whether the faces displayed angry or neutral expressions (more negative connectivity for angry faces), although this effect did not meet our criteria for significance. However, when the individual differences in reward–drive were taken into consideration, we found that the change in connectivity between the same regions (ventral ACC and amygdala) was highly correlated with reward–drive, ranging from more negative to less negative values with increasing reward–drive. DCM analysis supported and extended these findings by showing that this latter effect was restricted to connectivity from the ventral ACC to the amygdala, but not *vice versa*. Note also, that our results provide no evidence that connectivity between the amygdala and ventral ACC was modulated by the different measures of anxiety, other reward processing dimensions, or depression.

Our previous research showed that regional activation within the amygdala and ventral ACC in response to viewing angry (relative to neutral or sad) expressions is correlated with individual differences in reward–drive (Beaver et al., 2008), a dimension consistently linked to the tendency to display hostile and aggressive behaviour (Carver, 2004; Cooper et al., 2008; Cornell et al., 1996; Diefendorff and Mehta, 2007; Putman et al., 2004; Smits and Kuppens, 2005). The present study goes significantly further by showing that it is specifically variation in connectivity from the ventral ACC to amygdala, but not *vice versa,* that is affected by reward–drive. Variation that may explain why signals of aggression are interpreted as provocative by some individuals (high reward–drive) more than others.

The ventral ACC has been implicated in regulation of emotion. Hence, the reduced negative connectivity between the amygdala and the ventral ACC in high-relative to low reward–drive individuals, may represent a neuroanatomical marker of a reduced prefrontal control of amygdala function. Recently, Coccaro et al. (2007) studied individuals with intermittent explosive disorder, a psychiatric condition characterized by recurrent bursts of aggressive reactions. In contrast to controls, they demonstrated no connectivity between the amygdala and the ventromedial prefrontal cortex when exposed to aggressive displays (relative to fixation baseline). Taken together with the results of our own study this could represent a continuum in the function of ventromedial prefrontal cortex/ventral ACC regulation—ranging from optimal regulation in low reward–drive healthy individuals, to reduced regulation in high reward–drive healthy individuals, to dysfunctional regulation in psychiatric disorders of aggression.

## Reward–drive modulates connectivity from ACC to the amygdala

Although the nature of the DCM connectivity values is very different from GLM based PPIs, it is striking that the basic result was consistent—viewing angry relative to neutral faces produced a greater change in connectivity between the ventral ACC and amygdala. However, the DCM results go further by demonstrating that this effect is restricted to connectivity from the ventral ACC to amygdala but not *vice versa*, and that this was significantly correlated with reward–drive. It is of interest that this directionality accords with a recent and detailed anatomical study in monkeys (Ghashghaei et al., 2007), which demonstrated that the ventral ACC sends proportionally more projections to the amygdala than it receives; consistent with the ventral ACC's role in the extinction of negative emotions. Similarly, additional comparative research shows that the ventral ACC gates the transfer of the information in the amygdala from the basolateral nuclei (containing face-sensitive cells) to the central nucleus (involved in the expression of emotional arousal) by acting on GABA-ergic interneurons (Maren and Quirk, 2004; Rosenkranz and Grace, 2002). Previous research has shown that the pathway from ventral ACC to the amygdala is important in mediating the resolution of conflict in an emotional Stroop task (Etkin et al., 2006). However, whereas Etkin et al. showed that the right amygdala was involved in emotional conflict detection, our own study identified the left amygdala in processing signals of aggression. Our current findings are consistent with previous studies in both healthy and psychiatric individuals (intermittent explosive disorder) showing that the activation of the left amygdala (when viewing facial signals of aggression) was significantly modulated by reward–drive (Beaver et al., 2008) or by the severity of aggressive behaviour (Coccaro et al., 2007).

DCM also allowed us to test whether facial information is projected in parallel into the ventral ACC and amygdala, or whether the processing is serial (i.e., facial information enters the amygdala and is then projected to the ventral ACC, or *vice versa*). Previous animal and human literature provides evidence in favor of parallel inputs (Eimer and Holmes, 2007; Eimer et al., 2003; Etkin et al., 2006; Kawasaki et al., 2001; Leonard et al., 1985; Oya et al., 2002; Rolls, 2007). In particular, both the amygdala and ACC have robust and direct connections with secondary visual areas (e.g. posterior occipital cortex, fusiform gyrus, and superior temporal gyrus) involved in the perception of facial signals of emotion (Aggleton et al., 1980; Catani et al., 2002, 2003; McDonald and Mascagni, 1996). This sensory information is conveyed rapidly and influences the neuronal activity in the amygdala and prefrontal cortex at approximately the same time, as demonstrated by single cell recordings in monkeys showing similar short latencies for faces (∼ 110–220 ms) in both regions (Leonard et al., 1985; Rolls, 2007). Similarly, ERP experiments and in-depth implant electrode recording in humans have demonstrated rapid (∼ 150 ms) frontocentral potentials in response to emotional faces and scenes (Eimer and Holmes, 2007; Eimer et al., 2003; Kawasaki et al., 2001; Oya et al., 2002).

These results suggest that the rapid processing of emotional facial expressions within the anterior prefrontal cortex could occur independently and in parallel with processing in the amygdala. Consistent with this idea, Bayesian model selection (Penny et al., 2004) showed very strong evidence in favor of the parallel model (faces ‘injected’ simultaneously in both ventral ACC and amygdala) compared with serial models (faces ‘injected’ independently in either the amygdala or ventral ACC alone).

On the basis of our findings and previous research, we propose that the processing of facial signals of emotion by the amygdala and ventral ACC involves at least two stages. An initial input to both regions provides a rapid and coarse analysis of affective content. Following possible transfer of information from the amygdala to the ventral ACC, a more complex evaluative process of the socio-affective meaning of a facial expression in relation to the appropriate context and the individual's temperament and personality is implemented by the ventral ACC which projects the results of these computations to the amygdala; this may include gating the transfer of information between basolateral amygdala and central nucleus, as demonstrated by comparative research (Maren and Quirk, 2004; Rosenkranz and Grace, 2002). Of particular interest, it is this latter process that is captured by the anger-mediated effects of reward–drive from the ventral ACC to the amygdala.

fMRI does not have sufficient spatial resolution to detect specific amygdala nuclei. So we cannot be certain that ventral ACC afferents affect interactions between the basolateral and central nuclei of the amygdala, as opposed to other nuclei. However, as suggested by others (Aggleton, 1985; Emery et al., 2001; Stefanacci and Amaral, 2002), the basolateral amygdala is in an ideal position to act as a neural integrator that attempts to match an environmental emotional stimulus with a particular social context conveyed by the ventral ACC. Individual differences in the gating signal from the ventral ACC to the amygdala could therefore lead to differences in how an aggressive signal is perceived and interpreted, ultimately influencing the variability of behavioural reactions.

We have shown that the interactive effects of reward–drive and processing angry expressions in the amygdala and ventral ACC is found for contrasts comparing anger with neutral (current study) or other negative expressions (sadness) (Beaver et al., 2008). Moreover, there is little evidence that reward–drive personality affects the experience or perception of other emotions (i.e., happiness, fear, or sadness) (Carver, 2004; Putman et al., 2004). In view of these findings, we focused on angry and neutral expressions only. However, we do not wish to deny that the amygdala and ventral ACC have a wider role in emotional behaviour, and in emotion regulation in particular (Davidson et al., 2000; Drevets, 1999). The key role of the ventral ACC in controlling negative affects has been highlighted by paradigms testing fear extinction in both humans and animals (Delgado et al., 2006; LeDoux, 2003; Phelps et al., 2004) and human studies of anxiety suppression (Petrovic et al., 2005). Hence, our claim is that the reduced influence of viewing aggressive (compared with neutral) faces on the connectivity from the ventral ACC to amygdala (but not *vice versa*) in high reward–drive individuals, fits with the idea that emotion regulation plays a key role in aggression (Davidson et al., 2000). However, the influence of reward–drive on other negative emotions, such as disgust, whose recognition is also affected by aggressive disorders (Best et al., 2002), should be considered in future research.

A further consideration is that the angry and neutral expressions were presented in short blocks intermixed with null events. Although, this provided the optimal design for both PPI and DCM analyses, it might affect the predictability of emotional faces. Hence, a question for future research is whether a fully randomised event-related design might produce similar findings.

## Conclusions

We have demonstrated that connectivity from the ventral ACC to the amygdala is modulated by viewing facial signals of aggression, and that this effect is correlated with individual differences in reward–drive; a personality dimension linked to the tendency to display aggressive behaviour. At a group-based level, there was only a borderline change in connectivity when angry relative to neutral faces were presented. However, this was explained by the marked influence of variability in reward–drive on the connectivity between the amygdala and the ventral ACC. Our study identifies a highly specific locus for the interaction between reward–drive and processing signals of aggression and provides a potential neurobiological account for the influence of this personality dimension on aggression in general.

## Appendix A Supplementary data

Supplementary Table 1 Main effect of angry faces vs. baseline (n = 21, one sample t test on regional BOLD response).
