# Supplementary material for: Connectivity from the ventral anterior cingulate to the amygdala is modulated by appetitive motivation in response to facial signals of aggression
Source: Neuroimage. 2008 Nov 15;43(3):562–70. doi: 10.1016/j.neuroimage.2008.07.045 (PMC2581780; doi:10.1016/j.neuroimage.2008.07.045)
Supplement: Supplementary Table 1 — Main effect of angry faces vs. baseline (n = 21, one sample t test on regional BOLD response). [file mmc1.doc]

**Supplementary table 1.** Main effect of angry faces *vs*. baseline (*n* =21, one sample *t* test on regional BOLD response).

| **Cerebral Region** | **Side** | ***t***  **scores** | **MNI**  **coordinates**  **x y z** | | |
| --- | --- | --- | --- | --- | --- |
| **Amygdala** | **L** | 9.05 | -24 | -4 | -12 |
| **Putamen** | **L** | 8.40 | -20 | -4 | 8 |
| **Parahippocampal Gyrus** | **L** | 9.95 | -20 | -30 | -2 |
|  | **R** | 8.38 | 22 | -28 | -2 |
| **Inferior Frontal Gyrus** | **L** | 8.64 | -28 | 24 | 4 |
|  | **R** | 7.87 | 34 | 24 | 14 |
| **Dorsolateral Prefrontal Cortex** | **L** | 8.70 | -52 | 6 | 30 |
|  | **R** | 9.44 | 38 | 12 | 28 |
| **Supplementary Motor Cortex** | **L** | 9.10 | -6 | 0 | 56 |
|  | **R** | 7.73 | 6 | 12 | 52 |
| **Inferior Parietal Lobule** | **L** | 10.39 | -30 | -60 | 46 |
| **Fusiform Gyrus** | **L** | 14.90 | -36 | -54 | -20 |
|  | **R** | 16.47 | 28 | -52 | -26 |
| **Extrastriate Visual Cortex** | **L** | 11.59 | -38 | -80 | -8 |
|  | **R** | 19.60 | 36 | -86 | 0 |

**Table Footnotes:** FWE: Family Wise Error corrected, *p*<.05; L: left, R: right; MNI: Montreal Neurological Institute.
